# Risk-benefit analysis of emergency vaccine use

**DOI:** 10.1038/s41598-022-11374-7

**Published:** 2022-05-06

**Authors:** Gregory Lewis, Michael Bonsall

**Affiliations:** Department of Zoology, University of Oxford, Oxford, OX1 3SZ UK

**Keywords:** Vaccines, Public health, Health policy

## Abstract

Emergency vaccine use requires weighing a large number of uncertain risks and possible benefits. In the COVID-19 pandemic, decisions about what evidence is necessary to authorize emergency use have proven controversial, and vary between countries. We construct a simple mathematical model of the risks and benefits of emergency vaccination to an individual, and apply this to the hypothetical scenario of individual decision-making between emergency use of a COVID-19 vaccine without safety and efficacy data, versus waiting for efficacy and safety to be established. Even with conservative modelling assumptions and uncertainty distributions for vaccine efficacy (mean expectation = 17%) and serious adverse event risk (mean expectation = 0.3%), high risk individuals (e.g. those who are elderly and have a household contact with COVID-19) are better off using the ’emergency vaccine’ rather than waiting for more information (absolute risk reduction for mortality up to 2%). Very early emergency authorization of vaccines despite very limited data may be the better public health strategy when confronted with a dangerous emerging infectious disease.

## Introduction

Vaccination is rarely an emergency. Most vaccine candidates target an endemic and well-characterised infectious disease. In these cases, there is time to perform a rigorous vaccine candidate trial to establish a vaccine is safe and effective before it is offered for routine clinical use.

Emerging infectious diseases can be exceptions to this rule. For instance, in Guinea in 2016 and the DRC is 2017, Ebola vaccines still in phase 3 trials were used to combat Ebolavirus disease (EVD) outbreaks^1,2^. The ethical rationale was the risks of administering a vaccine which was not proven to be safe and effective was outweighed by the potential benefit of protection from a highly lethal infectious disease^3^. The WHO’s Emergency Use Listing (EUL) procedure, first developed in response to this EVD experience, is explicit about this trade-off^4^:

The EUL is a special procedure for unlicensed vaccines, medicines and in vitro diagnostics in the event of a PHE [Public Health Emergency] when the community/public health authorities may be willing to tolerate less certainty about the efficacy and safety of products, given the morbidity and/or mortality of the disease and the lack or paucity of treatment, diagnosis/detection or prevention options

The EUL also countenances emergency use of a vaccine candidate even when no human data on efficacy is yet available^4^:

If preliminary human data showing some efficacy are not available for the vaccine under consideration and if not imminently available for other vaccines being concurrently developed, WHO will consider whether the preponderance of evidence from the non-clinical, and early human studies justifies considering the immunogenicity data as a potential surrogate that is thought to be reasonably predictive of clinical efficacy.

Navigating these uncertainties are complex, as they typically apply not only to the vaccine candidate but to the emerging infectious disease itself: at the early stages of an outbreak, infection fatality rate, risks of long-term health consequences, infectiousness, and even mode(s) of transmission can be uncertain or unknown^5,6^. An individual offered emergency vaccination in such circumstances has to choose between two very unclear risks: whether, in their situation, the risks of the vaccine are greater or less than those of remaining susceptible to the disease.

For policy-makers deciding on emergency use authorisation, the benefit/risk to the individual is further complicated by population-level risks and benefits. One complex decision would be, if the vaccine impedes transmission, earlier use could lead to greater herd immunity, so reducing the final size of the epidemic^7^; another would be the risk of, if the vaccine is less safe and effective than hoped, damaging public confidence in subsequent vaccination campaigns for the diseases, and potentially vaccination programs for other diseases as well^8–10^.

The COVID-19 pandemic has brought these challenges to global attention. Regulatory practices varied between countries for vaccine licensure. Many regions (e.g. US, UK, Australia, Europe) accepted interim phase 3 trial results as sufficient for a vaccine candidate to be deployed, but their requirements differed: some needed to see an early signal of efficacy across the population, sometimes with a threshold of at least 50% vaccine efficacy; whilst others required efficacy and safety data specifically for subgroups ’first in line’ for administration. Both China^11^ and Russia^12^ have offered their vaccine to their population or for international sale before phase 3 results were available.

All approaches, whether more aggressive or more conservative, have proven controversial. Although most candidate vaccines now have extensive clinical data available, the dilemmas around emergency use remain live. These issues include: how should regulation treat modified existing vaccines (or modified schedules of existing vaccine administration) addressed to deal with emerging COVID-19 strains; how can new vaccine candidates which may reduce global COVID-19 vaccine shortfall be deployed; and what are the best approaches for constructing policy for future emerging diseases.

These challenges of decision-making under uncertainty are widely appreciated in medicine and public health^13–15^. The dilemma of emergency vaccine use—whether to act now on limited knowledge, or wait for more information to become available - is similar to problems about health economics in assessing the value of further information in medical research or decision support^16,17^. Although mathematical evaluation of all ramifications of emergency vaccine use may be intractable, analyses restricted to individual risk-benefit can be informative: if individuals should expect significant benefit or harm if they elected for emergency vaccination, this argues strongly for or against it being offered in the first place.

This paper attempts this evaluation. We first construct a simple mathematical framework of individual risk-benefit for vaccine use. We then adapt this for emergency vaccination for the COVID-19 pandemic by using epidemiological parameter ranges of this pandemic alongside vaccine safety and efficacy track records. Our modelling then explores the scenario of an individual being offered an emergency vaccination for COVID-19, where the vaccine candidate has demonstrated encouraging early results, but the phase 3 trial is ongoing and interim data is not yet available. This situation allows us to assess an approach similar to that used in China and Russia, and arguably different than used in most other countries.

## Methods

### A model of vaccine risk-benefit

We use the following equation to evaluate the risks and benefits to an individual from being vaccinated:

1 $$\begin{aligned} \text {Utility} = \text {P(I)} \cdot \text {IFR} \cdot \text {VE} - \text {SAER} \end{aligned}$$

Where P(I) is the probability of infection, IFR is the infection fatality rate (i.e. $\text {p(Death|Infection)}$), VE is the vaccine efficacy, and SAER is the serious adverse event risk. The first three terms give the benefit in terms of mortality risk reduction from the disease; the last term gives the risk increase accrued through vaccination. Utility $\le 0$ if any of the first three terms are zero: one cannot benefit from vaccination where one will certainly not acquire the infection, or certainly not die from the infection, or where the vaccine is certain to confer no protection.

This equation underestimates the net-benefit of vaccination in two respects. First, many infections (including COVID-19) can result in significant morbidity even they do not kill, but potential benefits from vaccination in avoiding these other sequelae are not included. Second, the great majority of serious adverse events in vaccines, whether clinical or experimental, are not fatal, yet equation 1 treats these events as equivalent to dying from the disease.

### Emergency use

We consider a simplified scenario of an individual being offered an emergency vaccine whose safety and efficacy are currently unknown, but will be discovered subsequently. For concreteness, this could correspond to an individual being offered a vaccine like Pfizer/BioNtech after the publication of the phase I/II results (in August 2020), but prior to interim Phase III data (reported 4 months later in December). We compare two choices:

1. **Go.** The individual takes the vaccine early, taking an uncertain risk from vaccination for the uncertain benefit of potential earlier protection from the disease.
2. **Wait.** The individual waits for firm data on vaccine safety and efficacy, taking an uncertain risk from remaining fully susceptible to the disease until this is established.

We now adapt equation 1 to compare ‘Go’ versus ‘Wait’.

2 $$\begin{aligned} \text {Expected Utility} = \widehat{\text {P(I)}}_{(t(e), t(k))} \cdot \widehat{\text {IFR}} \cdot \widehat{\text {VE}} - \widehat{\text {SAER}} \end{aligned}$$

If the Expected Utility is positive, then emergency use is superior to waiting, and vice versa. The first change is this equation now represents a forecast rather than a point calculation of risk. The expected utility is a function of the expected values of P(I), IFR, VE, and SAER. The second change is greater precision on what is meant by ‘probability of infection’: an individual who opts not to take an emergency vaccine, preferring to wait for a vaccine which is firmly established to be safe and effective, is not deciding to remain susceptible to infection permanently. The risk of infection is therefore the probability of infection in the interval between the time they could have taken the vaccine as an emergency (*t*(*e*)) and the time when the vaccine’s safety and efficacy are known (*t*(*k*)). (For brevity, this will be simply ‘P(I)’).

This approach is also conservative. The serious adverse event risk would still apply if the vaccine was taken at *t*(*e*) instead of *t*(*k*). This risk is only a penalty of emergency use for individuals who elect for the vaccine at *t*(*e*) but who subsequently (at *t*(*k*)) have better options than this vaccine available to them.

### Application to COVID-19

We now consider a parameterization of equation 2 in light of the COVID-19 pandemic (Table 1).

**Table 1:** COVID-19 vaccine emergency use modelling parameters.

Variable	Values
Probability of Infection (p(I))	*Range*: 0 to 0.4
Infection Fatality Rate (IFR)	*Range*: 0 to 0.2
Vaccine Efficacy (VE)	*Distribution* Conservative: $p = 1/3: \text {VE} = 0$; $p = 2/3: \text {VE} = Beta(1,3)$ Optimistic: Uniform(0,1)
Serious Adverse Event Risk (SAER)	*Distribution* Conservative: *Beta*(1, 301) Optimistic: log-uniform ($10^{-6}$, $10^{-3}$)

IFR and probability for infection over a given period for COVID-19 can vary dramatically between individuals: a twenty year old woman living alone in China may have an $\widehat{\text {P(I)}}$ and $\widehat{\text {IFR}}$ three orders of magnitude lower than an 80 year old man who becomes a household contact of an active case. Given this variation, we use plausible ranges rather than point estimates. For $\widehat{\text {P(I)}}$, we consider the range (0, 0.4) the lower limit approximating someone living in a very low incidence environment (e.g. New Zealand, which had a total cumulative COVID-19 incidence of $\approx$ 35 per 100 000 n 2020^18^ i), the upper limit in the region of estimates of secondary attack rate for household contacts^19^. For $\widehat{\text {IFR}}$, we consider the range (0, 0.2), corresponding to the range of age-specific infection fatality rates estimated by Brazeau and colleagues^20^.

SAER and VE have less dramatic between-individual variation, but the ‘typical’ true value of VE and SAER of an unproven vaccine are uncertain. We consider the random variables $\widehat{\text {SAER}}$ and $\widehat{\text {VE}}$, estimators of VE and SAER, and construct both ‘conservative’ and ‘optimistic’ distributions for each.

For $\widehat{\text {SAER}}$, our conservative estimator ($\widehat{\text {SAER}}_c$) is based on the rate of vaccine-attributed serious adverse events observed in earlier studies of the vaccine candidate. For vaccines in phase 3 trials, the typical number of adverse events previously observed in phase 1 and 2 trials are zero. We use a conservative prior (*Beta*(1, 1)) and update this based on *N* consecutive observations of no serious adverse event ($Beta(1, 1 + N)$). We use $N=300$, thus a conservative uncertainty distribution of *Beta*(1, 301), with an expected value (${\text {E}}[\widehat{\text {SAER}}_c]$) of roughly 0.3%. This is consistent with the rates of adverse events seen in Phase 1 studies, reviewed by Johnson and colleagues^21^. They found 15 serious adverse events attributable to the agent being trialled in 24988 participants in the treatment arms of studies reviewed. They also report vaccine trials had a higher rate than other study types (incident ratio of 6.21). This gives ${\text {E}}[\widehat{\text {SAER}}_c] \approx 0.4\%$.

This approach is conservative as $\widehat{\text {SAER}}_c$ does not use the knowledge that vaccines are typically much safer, with serious adverse event rates typically 1/10000 or less, and that vaccine candidates which reach later trials are selected for safety out of all candidates tested in earlier stages. Our ‘optimistic’ estimator ($\widehat{\text {SAER}}_o$) presumes the vaccine candidate is similarly safe to vaccines already used in routine practice. As adverse event rates for these vaccines can vary from one in millions to one in thousands, we use a log-uniform distribution from ($10^{-6}$, $10^{-3}$).

For $\widehat{\text {VE}}$, our ‘optimistic’ estimator ($\widehat{\text {VE}}_o$) is the maximum entropy distribution for the interval (0, 1), the uniform distribution: we do not believe any given value for vaccine efficacy is more or less likely than any other; ${\text {E}}[\widehat{\text {VE}}_o] = 0.5$. Our ‘conservative’ estimator ($\widehat{\text {VE}}_c$) is more pessimistic in two respects: first, there is a significant probability the vaccine candidate is completely ineffective (for Uniform(0,1), VE is almost surely $> 0$), and it is more likely to have poor efficacy (VE $< 0.5$) than good efficacy. We therefore use a mixed distribution where one third of the probability mass is VE $= 0$, and the remainder is distributed by *Beta*(1, 3); ${\text {E}}\widehat{[\text {VE}}_c] \approx 0.17$.

### Analysis

We model our emergency vaccine use scenario for COVID-19 by dividing the plausible range of P(I) and IFR into 21 equidistant points (i.e. for IFR: 0, 0.01, 0.02 ... 0.2; for P(I): 0, 0.02, 0.04 ... 0.4.). For each of these 441 combinations of (P(I), IFR), we draw values at random from an estimator of VE and SAER, and calculate the utility. We repeat this 10000 times and take the average to give the expected value.

We also take the proportion of samples with Utility> 0 to give a ‘likelihood of expected benefit’: the probability individuals at a given value of (P(I), IFR) would find they were better off in expectation for electing for emergency vaccination when the true values of SAER and VE are known. This is distinct from a straightforward ’likelihood of overall benefit’. For example, emergency use of a vaccine with VE = 0.1 and SAER = 0 is certain to provide expected benefit to any individual for whom P(I) and IFR > 0: this vaccine cannot harm, and may help should they be exposed to the infection. Yet the likelihood of benefit of taking the vaccine cannot be greater than the Vaccine Efficacy (0.1).

### Data and code

Analysis was conducted on MATLAB (v. R2020a; Mathworks, Massachusetts). All code and data is available at: https://github.com/gjlewis37/EUAVax/.

## Results

### Surfaces of equipoise

**Figure 1:**
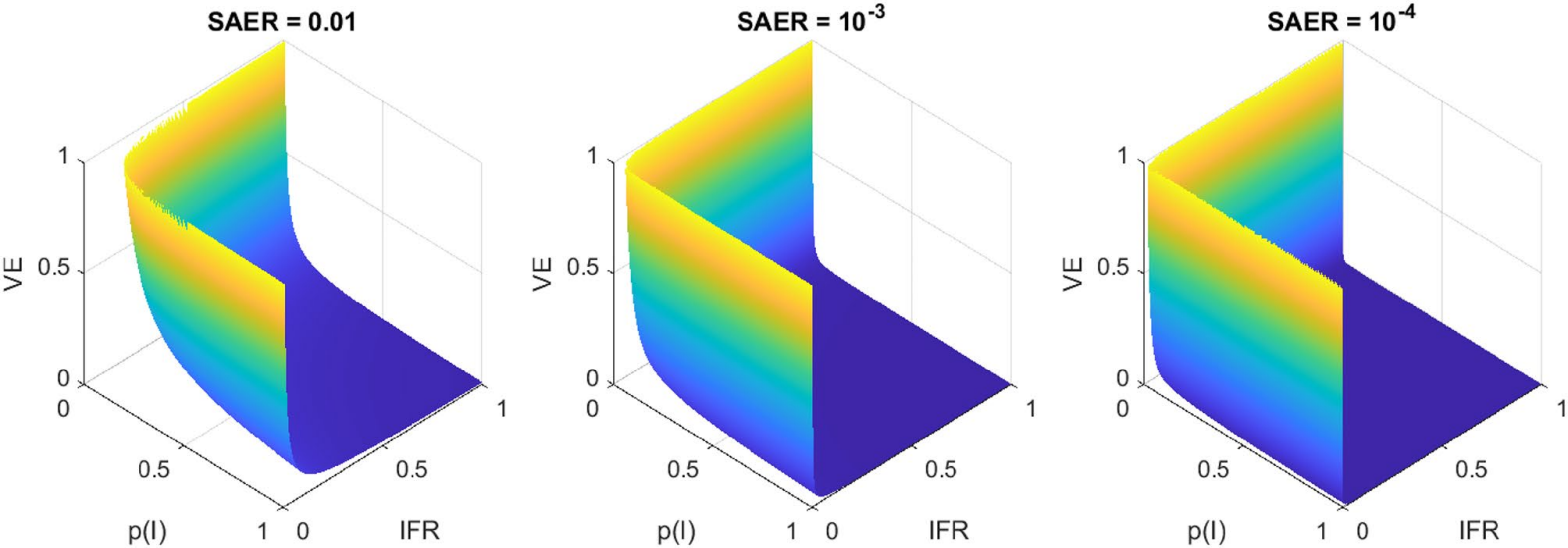
Surfaces of clinical equipoise for vaccination (Utility = 0) in terms of probability of infection (P(I)), Infection Fatality Rate (IFR) and Vaccine Efficacy (VE) for Serious Adverse Event Rate (SAER) = 0.01 (l), $10^{-3}$ (c), and $10^{-4}$ (r). The volume above this surface is where Utility > 0, thus vaccination is beneficial, and vice versa.

For a given value of SAER, equation 1 produces a surface of equipoise which is a three dimensional hyperbola with respect to P(I), IFR, and VE (Fig. 1). The threshold value for VE falls with increasing values of P(I) or IFR: intuitively, compensating for a given harm of vaccination (SAER) can be achieved by a greater risk reduction from a less dangerous disease, or a lesser risk reduction from a more dangerous one. Decreasing SAER in essence lowers this constraint, with correspondingly more of the unit volume above the surface of equipoise - that is, where vaccination would be beneficial.

**Figure 2:**
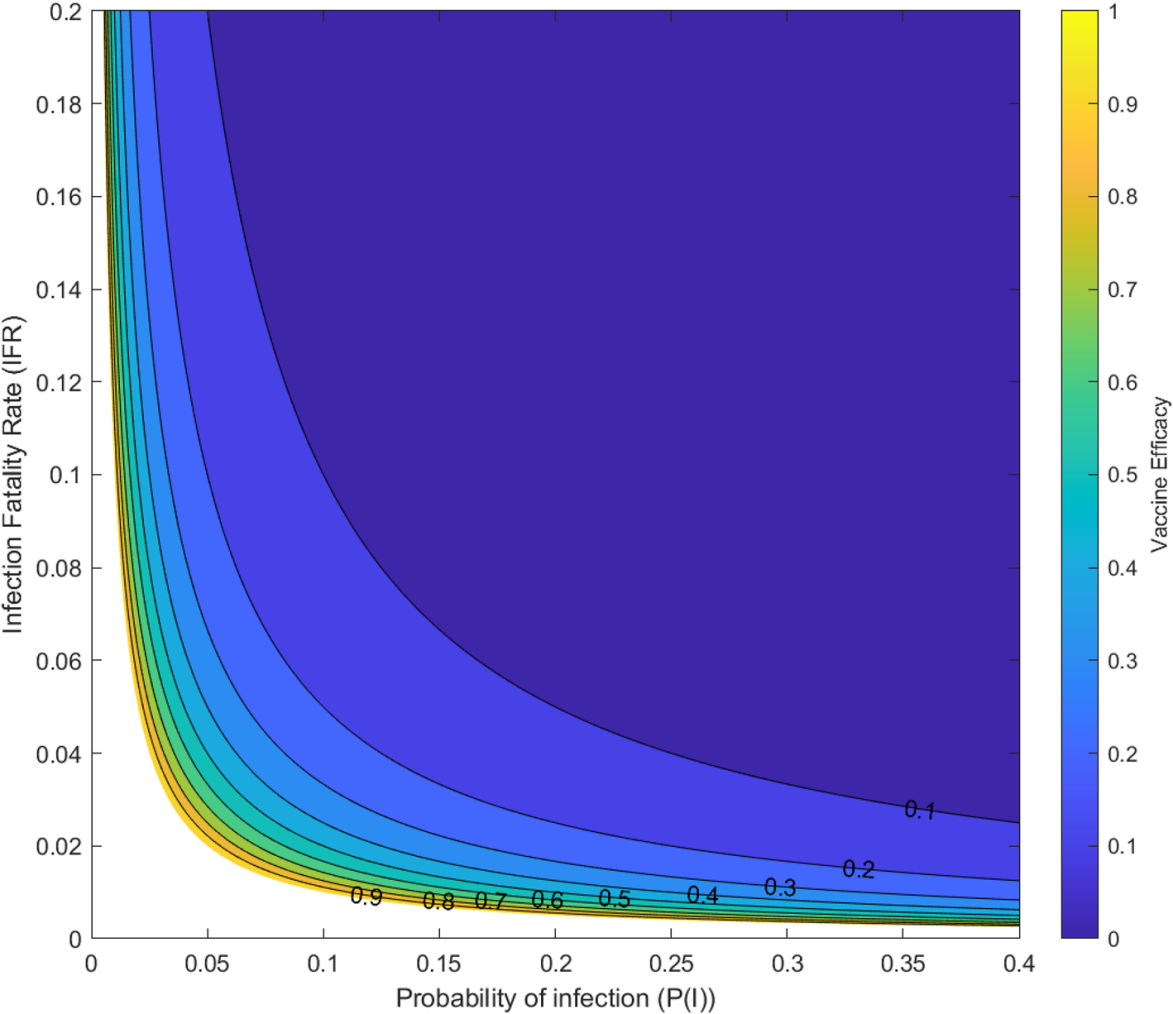
Contour plot of Vaccine Efficacy (VE) versus probability of infection (P(I)) and Infection Fatality Rate (IFR), for ranges of these parameters relevant to COVID-19 ($0 \le \text {P(I)} \le 0.4$; $0 \le \text {IFR} \le 0.2$), given a Serious Adverse Event Rate (SAER) of 0.1%. The VE = 0.1 contour includes more than half of this parameter region, and so Utility > 0 for a vaccine efficacy of 10% in this region of values. Further increments of VE include progressively smaller additional proportions of this field of (P(I), IFR). Note the unshaded region is where vaccination is not beneficial even with a perfectly effective vaccine, given by $0.001 \ge \text {P(I)} \cdot \text {IFR}$: this is the region where the risk of disease is lower than than the SAER of the vaccine.

This also means for a given SAER, the marginal returns to increasing VE in terms of the area of (P(I), IFR) where vaccination is beneficial are decreasing. The central case, SAER = $10^{-3}$, is illustrated in a contour plot with IFR and AR restricted to the parameter ranges relevant to COVID-19: $0 \le \text {P(I)} \le 0.4$; $0 \le \text {IFR} \le 0.2$ (Fig. 2).

### Risk-benefit of emergency COVID-19 vaccination

**Figure 3:**
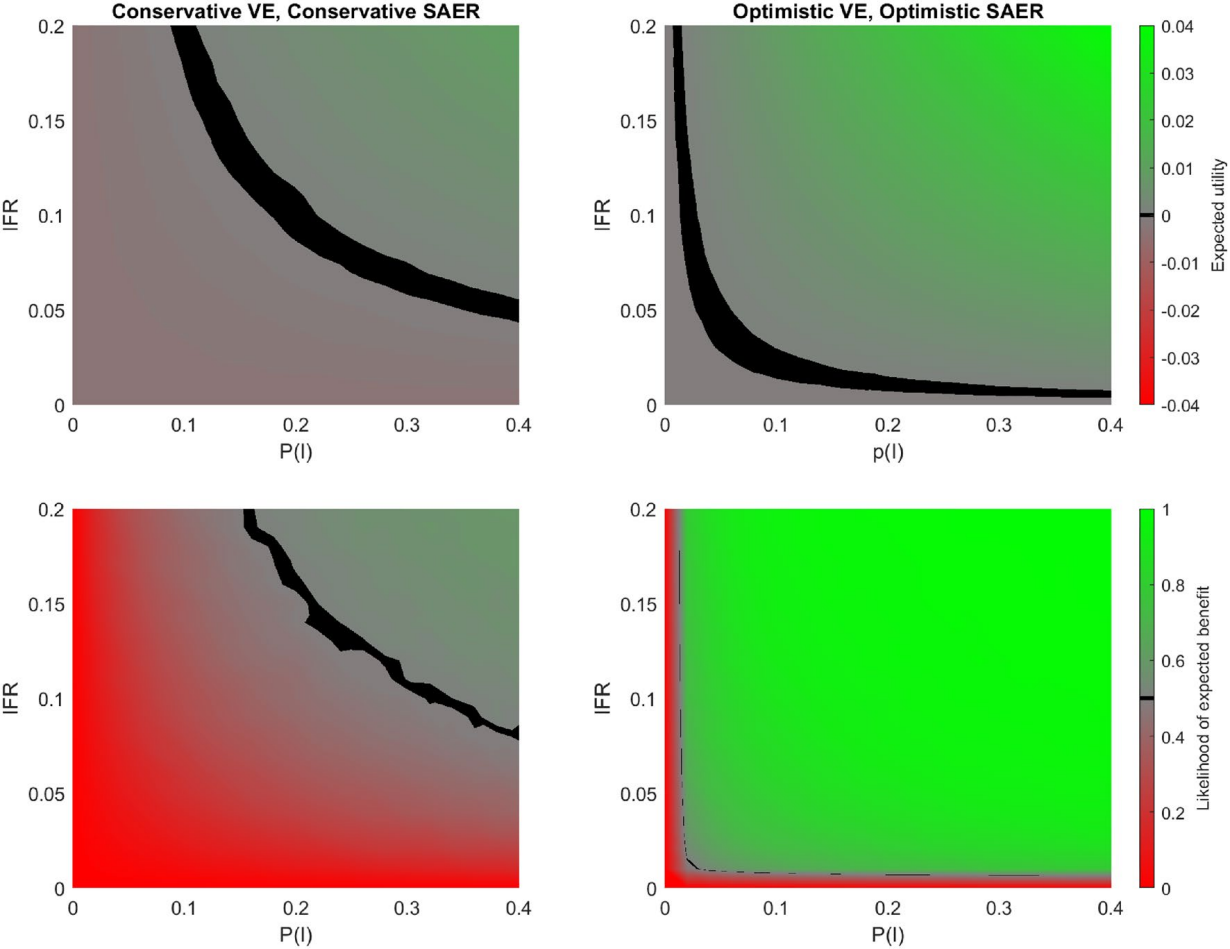
Expected utility (top row) and likelihood of expected benefit (bottom row), for the model of emergency COVID-19 vaccination with conservative (left column) and optimistic (right column) estimators for VE and SAER. Green denotes positive utility or likelihood of expected benefit > 0.5, and red the opposite. The black region corresponds to that of approximate equipoise between emergency vaccination or not: $-0.0005<$ Expected Utility $\le 0.0005$ and $0.495<$ Likelihood of expected benefit $\le 0.505$ respectively. The optimistic estimators result in emergency vaccination being beneficial across a greater proportion of the [P(I),IFR] parameter space. However, with either conservative or conservative expectations, those at high risk of COVID-19 - the top right region of these graphs - can expect significant benefit.

The model for risk benefit in emergency vaccination for COVID-19 is given in Fig. 3. Notably, even with conservative estimators for both VE and SAER, individuals subject to higher P(I) and IFR are expected to benefit from emergency vaccine use. Those at lower risk of infection (and death conditional on infection) are more likely to be harmed by emergency vaccination, but this expected harm is relatively low (at most $\approx -0.003$, matching ${\text {E}}[\widehat{\text {SAER}}_c]$). In contrast, the benefit of those at greatest risk are much greater: at the extreme of P(I) = 0.4, IFR = 0.2, and with ‘conservative’ estimators for VE and SAER the expected utility is $\approx 0.02$. As the units for utility is probability of survival, this amounts to an absolute mortality risk reduction of 2%, thus a number needed to vaccinate of $\frac{1}{\text {Absolute risk reduction}} \approx 50$.

With more optimistic estimators for both VE and SAER, emergency vaccination offers both greater expected benefit and higher likelihood of net benefit across the range of P(I) and IFR (e.g. at p(I) = 0.4 and IFR = 0.2, the absolute risk reduction is $\approx 0.04$). Emergency vaccination is consequently beneficial to individuals across a wider range of (P(I), IFR) values. The modelling scenarios of conservative estimators for VE alongside optimistic expectations for SAER (and vice versa) give results intermediate between those shown in Fig. 3, (Figure S1, supplementary information).

Likelihood of expected benefit shows a similar pattern to simple expected benefit, although the thresholds for equipoise in terms of (P(I), IFR) are lower, especially with ’optimistic’ estimators for VE and SAER. This is owed to distributions for SAER being highly skewed, with the median or modal SAER being lower than the mean. Thus in regions of neutral expected benefit the majority of samples are positive expected value, with the minority distributed in a longer tail below zero.

## Discussion

Our mathematical analysis suggests that even relatively ineffective vaccines can be beneficial when the danger from disease is high enough: the benefit of a 10% risk reduction from a highly contagious and lethal infectious disease can outweigh the risks of vaccination, even if these are (by vaccine standards) relatively great. The hyperbolic nature of the relationships between net-benefit and VE also means, for a given SAER, that the thresholds for net benefit (in terms of P(I) and IFR) fall further when moving from VE of 0.1 to 0.2 than from 0.5 to 0.9.

When we apply this model to emergency vaccination for COVID-19, ‘gambling’ on an uncertain vaccine can be the safer bet than accepting the dangers of prolonged susceptibility for those at high risk of COVID-19. This result occurs despite a very conservative approach in assessing the risks and benefits, and with conservative expectations of the emergency vaccine’s safety and efficacy. With more optimistic expectations, both the magnitude of benefit and the range of (P(I),IFR) where emergency vaccination is expected to be beneficial increase.

Our model makes many simplifying assumptions. We assume no interactions between variables, despite older individuals (a greater IFR) often mount less effective vaccine responses (a lower VE)^22^. An individual’s ‘Utility’ may not simply be mortality risk-reduction: individuals may put particular weight on particular health states which may be more or less likely to result from disease or vaccination. We also ignore relatively minor costs of both vaccination and infection, such as the inconvenience of getting a vaccination, pain of injection, or an infection which gives a mild illness and complete recovery.

Our modelling scenario is a highly simplified ‘snapshot’ scenario, so neglects many of the dynamics which could attend emergency vaccine use in an evolving pandemic. Examples of these include: (1) The outcomes for individuals who go ahead with emergency vaccination may generate observational data to improve initial estimates of vaccine efficacy and safety for those making decisions subsequently; (2) IFR and P(I) may not only be uncertain but non-stationary (e.g. new variants or therapeutic breakthroughs may emerge); (3) P(I) itself may be sensitive to how widely emergency vaccination is offered and accepted.

Our model for COVID-19 does not account for many vaccine candidates being developed in parallel. Although our model is indifferent to an individual who waits for the safety and efficacy of the emergency vaccine to be known or for another proven vaccine to become available, we do not model the possible risk of ‘vaccine-vaccine interference’^23^. Emergency use of a relatively ineffective vaccine could compromise the protection the individual gains from being subsequently administered a more effective vaccine.

Our results are highly sensitive to the presumed distributions of $\widehat{\text {VE}}$ and $\widehat{\text {SAER}}$. Our ‘conservative’ estimators may not be conservative enough. However, we note that the three earliest COVID-19 vaccine candidates reported interim results with VE > 0.5 (Pfizer/BioNtech^24^, Moderna^25^, Oxford/Astrazenica^26^). These are a very surprising conditioned on our model of conservative expectations for VE (p < 0.001) - even more conservative expectations would find this outcome even more improbable. Our ‘optimistic’ uncertainty distribution for VE is less surprised by these results (p = 0.125), suggesting it is a more reasonable prior. Similarly, the combined SAERs for these vaccines are much lower than 0.3% (e.g. $\approx 0.0002\%$ of thrombotic events following Oxford/Astrazenica vaccination^27^, $\approx 0.0001\%$ of myocarditis or pericarditis following Moderna or Pfizer/BioNtech vaccination^28^), and much more in the range of ’one in thousands’ to ’one in millions’ given by our ‘optimistic’ estimator. Wong, Siah and Lo, reviewing previous trials, find the likelihood of a vaccine candidates in phase 1 studies ultimately reaching approval for use is 33.4%, rising to 85.4% if the candidate reaches phase 3 trials^29^. This suggests a given vaccine candidate being subsequently found safe and effective is much more common than our conservative assumptions imply.

Much more significant is our model does not account for the possibility of vaccine-dependent enhancement of disease (VDE)^30,31^. Loosely, this can be thought of as a VE which is less than zero. Modelling this is particularly complex as the expected likelihood and degree of VDE either generally or in a particular scenario remains very poorly understood. However, this risk only qualitatively changes the results when it is so high the expected VE falls to very low values. In the conservative model, the highest risk individuals only cease to benefit in expectation from emergency vaccination when the expected vaccine efficacy is $\approx 0.04$ or less: a vaccine scarcely more likely to protect rather than enhance disease.

One should be cautious using models like these to guide individual decision-making around emergency use. For example, although age is the most significant predictor for COVID-19 IFR, a given individual’s IFR would also depend on a number of other factors^32–34^. Similarly, an individual’s P(I) depends on the time remaining until safety and efficacy is firmly established, the incidence in their environment (which can change over this time), and their own behaviour. All are challenging to assess.

Two misinterpretations are important to guard against. The first is although our modelling shows large proportions of the (P(I), IFR) parameter space favours emergency use, this does not mean most individuals in a population stand to benefit from it. Both P(I) and IFR are highly skewed distributions across individuals: although some would find themselves in the ‘top right corner’ (e.g. elderly individuals with an infectious household contact), most would find themselves closer to the bottom left. A P(I) of 1% is in the range of seroconversion estimates over 3 months in countries with poorly controlled epidemics; the IFR generally only begins to exceed 1% in those over 60 years of age^20^. Insofar as our model implies a recommendation for emergency use, it only applies to fraction of the population at high risk, similar to those already assessed as highest-priority for vaccination under most vaccination deployment strategies.

The second is that individuals our model assesses as not benefiting from emergency vaccination would also not benefit from non-emergency vaccination. Our model only considers the benefit of potential short term protection (e.g. the interval between phase 2 and interim phase 3 data) in the context of uncertainty around VE and SAER. For non-emergency use, VE and SAER would be known much better, and the overall risk-benefit would need to consider a much longer duration of protection (which may be lifelong), the probability of infection over this much longer period, and that an individual’s IFR increases as they age. Our model does not apply to this scenario.

Our model only assesses risk-benefit to the individual, and so does not include population-level factors such as the potential of risk compensation, herd immunity, or the potential of emergency use to enhance or undermine public confidence in vaccination. These factors are important to policy-makers contemplating whether to authorize emergency use, yet they are difficult to assess and weigh alongside the individual-level benefits to estimate the socially optimal policy. We hope mathematically articulating the risk-benefit for individuals can inform these difficult decisions; on the face of it, the significant expected benefit at an individual level for those at high risk would require a lot to outweigh these gains.

Our mathematical analysis underlines that risk reduction can involve trade-offs, and calculation cannot be done purely in qualitative terms of ‘un/safe’ or ‘in/effective’. When one faces little risk of infection with a mild disease, the benefits of vaccination may not be worth even remote risks of harm. The opposite is also true: if confronted with a high likelihood of infection by a highly lethal pathogen, a vaccine which is ‘ineffective’ (e.g. a VE much lower than 50%) and - by vaccine standards - very ‘unsafe’ (e.g. an SAER of 1%) could still be better than nothing.

We also note the rationale for a ’50%’ efficacy threshold appears dubious. Not only would a hypothetical vaccine of (e.g.) 15% efficacy and a reasonable safety profile be better than nothing for many individuals, but such a threshold would rule out the great majority of primary and secondary prevention if it was applied to non-communicable disease: among many examples, the risk reduction of anti-hypertensives for coronary heart disease and stroke is $\approx 20\%$ and $\approx 40\%$ respectively, statin medication on cardiovascular disease is $\approx 25\%$^35^, and aspirin $\approx 20\%$^36^ for secondary prevention of vascular disease. All of these medications also have significant risks of adverse events: for example, $\approx 0.1\%$ of major gastrointestinal bleeds each year of aspirin use^37^.

COVID-19 will not be the last pandemic. The success of COVID-19 vaccine development demonstrates the potential for accelerated vaccine deployment to protect the global population from the next one. For this pandemic, the timelines for trials roughly matched those for manufacturing and logistics; the global vaccine shortage for vaccines has and will persist long after the clinical trials have concluded. Manufacturing and deployment will hopefully further improve between now and the next pandemic. If they do, data and decision-making may become the rate-limiting step to vaccine deployment.

There are a number of proposals to make this step faster, from adopting optimal adaptive trial design^38^, to ‘pre-positioning’ relevant pre-clinical work in advance of the emergence of a new infectious disease^39^, to the use of human challenge trials to rapidly find early signals of efficacy^38,40^. Another significant advantage of human challenge trials is the rapid potential to detect vaccine enhancement of disease.

Our work suggests a complementary line of research is to improve decision-making where uncertainty remains—whether a given situation warrants hasty action or watchful waiting. Prediction platforms^41^, ‘superforecasters’ and markets all shifted in response to pre-clinical COVID-19 vaccine data, implying this information was being used to predict whether particular vaccine candidates would ultimately prove effective. Attempting these forecasts explicitly, and assessing their accuracy and reliability could provide an important decision aid for emergency use policy under uncertainty.

One key goal to aid this capability, still to be pioneered, would be approaches to find general ‘base rates’ for vaccines: e.g. how effective is a vaccine candidate likely to prove given particular results from phase 2 (or phase 1) studies; what is the typical risk of a vaccine candidate provoking VDE; whether one vaccine modality tends to prove more or less effective than others, and so forth.

Ultimately, our modelling underlines that uncertainty may not always justify delay. ‘Gambling on an unproven vaccine’ may be safer bet for an individual than ‘gambling on not being infected while waiting for the vaccine to be proven’. In COVID-19, the cost of the latter can be stark - at the extreme of risk, a 1-4% absolute risk of death. The underlying driver for these results is that vaccines, even experimental ones, are very safe; remaining susceptible to COVID-19, for some, is extremely dangerous. With the benefit of hindsight, delaying administration of vaccines subsequently shown to be safe and effective has cost lives. Our work suggests the same could have been recognised in advance.

## Supplementary Information

Supplementary Information.

## Data Availability

All analysis and figure generation code is available at: https://github.com/gjlewis37/EUAVax/.
