# Supplementary material for: Risk-benefit analysis of emergency vaccine use
Source: Sci Rep. 2022 May 6;12:7444. doi: 10.1038/s41598-022-11374-7 (PMC9076899; doi:10.1038/s41598-022-11374-7)
Supplement: Supplementary file 1 — Supplementary Information. [file 41598_2022_11374_MOESM1_ESM.pdf]

## Supplementary Information

The modelling results for risk-benefit with an optimistic estimator for VE, but a conservative estimator for SAER (and vice-versa) are given in figure S1.

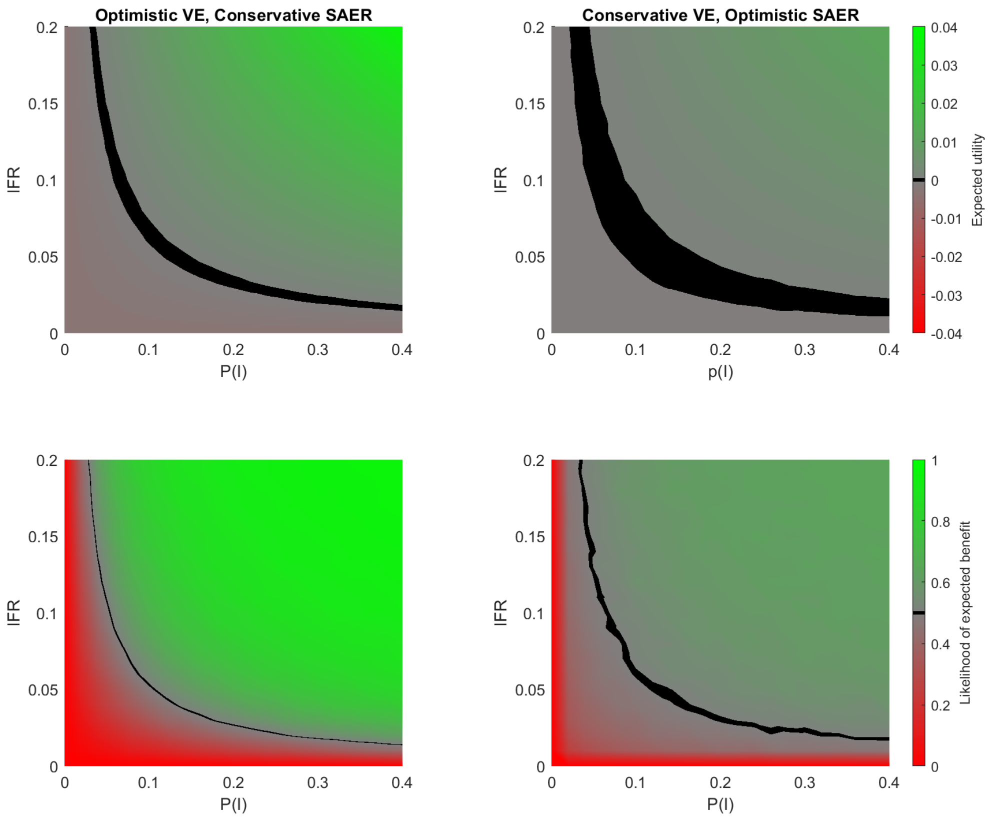


**Figure S 1.** Expected utility (top row) and likelihood of expected benefit (bottom row), for the model of emergency COVID-19 vaccination with the optimistic estimator for VE and the conservative one for SAER (left column) and vice-versa (right column). Green denotes positive utility or likelihood of expected benefit > 0.5, and red the opposite. The black region corresponds to that of approximate equipoise between emergency vaccination or not: $-0.0005 <$ Expected Utility $\leq 0.0005$ and $0.495 <$ Likelihood of expected benefit $\leq 0.505$ respectively. These results are approximately intermediate between the modelling where conservative or optimistic estimators are used for both VE and SAER (see figure 3).
